# Differential Modulation of Dorsal Raphe Serotonergic Activity in Rat Brain by the Infralimbic and Prelimbic Cortices

**DOI:** 10.3390/ijms24054891

**Published:** 2023-03-03

**Authors:** Elena López-Terrones, Verónica Paz, Leticia Campa, Sara Conde-Berriozabal, Mercè Masana, Francesc Artigas, Maurizio S. Riga

**Affiliations:** 1 Departamento de Neurociències i Terapèutica Experimental, Institut d’Investigacions Biomèdiques de Barcelona (IIBB-CSIC), 08036 Barcelona, Spain; 2 Centro de Investigación Biomédica en Red de Salud Mental (CIBERSAM), Instituto de Salud Carlos III, 28029 Madrid, Spain; 3 Departamento de Biomedicina, Institut de Neurociènces, Facultat de Medicina i Ciències de la Salut, Universitat de Barcelona, 08036 Barcelona, Spain; 4 Institut d’Investigacions Biomèdiques August Pi i Sunyer (IDIBAPS), 08036 Barcelona, Spain; 5 Centro de Investigación Biomédica en Red Sobre Enfermedades Neurodegenerativas (CIBERNED), 08036 Barcelona, Spain; 6 Centro Andaluz de Biología Molecular y Medicina Regenerativa (CABIMER-CSIC), 41092 Sevilla, Spain

**Keywords:** infralimbic cortex, prelimbic cortex, 5-HT neurons, in vivo electrophysiology, intracerebral microdialysis

## Abstract

The reciprocal connectivity between the medial prefrontal cortex (mPFC) and the dorsal raphe nucleus (DR) is involved in mood control and resilience to stress. The infralimbic subdivision (IL) of the mPFC is the rodent equivalent of the ventral anterior cingulate cortex, which is intimately related to the pathophysiology/treatment of major depressive disorder (MDD). Boosting excitatory neurotransmission in the IL—but not in the prelimbic cortex, PrL—evokes depressive-like or antidepressant-like behaviors in rodents, which are associated with changes in serotonergic (5-HT) neurotransmission. We therefore examined the control of 5-HT activity by both of the mPFC subdivisions in anesthetized rats. The electrical stimulation of IL and PrL at 0.9 Hz comparably inhibited 5-HT neurons (53% vs. 48%, respectively). However, stimulation at higher frequencies (10–20 Hz) revealed a greater proportion of 5-HT neurons sensitive to IL than to PrL stimulation (86% vs. 59%, at 20 Hz, respectively), together with a differential involvement of GABA_A_ (but not 5-HT_1A_) receptors. Likewise, electrical and optogenetic stimulation of IL and PrL enhanced 5-HT release in DR in a frequency-dependent manner, with greater elevations after IL stimulation at 20 Hz. Hence, IL and PrL differentially control serotonergic activity, with an apparent superior role of IL, an observation that may help to clarify the brain circuits involved in MDD.

## 1. Introduction

The prefrontal cortex (PFC) is the highest-level association cortex. It is dedicated to the representation, planning and execution of actions under a temporal pattern and is strongly involved in - higher brain functions, such as perception, attention, memory, language, intelligence, consciousness, affect, etc., many of which are altered in neuropsychiatric disorders. In particular, the dorsolateral PFC (e.g., Brodmann area 46) plays a key role in cognitive processes such as working memory and executive functions [1,2,3,4]. In turn, the ventral anterior cingulate cortex—vACC (e.g., Broadmann area 25)—is associated with the processing of emotional information [5,6,7,8,9]. The PFC displays extensive reciprocal connectivity with most cortical and subcortical structures, except for the basal ganglia, which feedback to the PFC through the thalamus. This connectivity confers the PFC the highest integrative level of all cortical areas and enables it to exert a “top-down” control of behavior by selecting one of the internally represented possible scenarios [1,4].

In particular, the PFC reciprocally connects with the monoaminergic nuclei (raphe nuclei, 5-hydroxytryptamine or serotonin (5-HT); locus coeruleus, noradrenaline (NA); ventral tegmental area, dopamine (DA)), modulating their activity [7,9,10,11,12,13,14,15,16,17,18,19]. Interestingly, the PFC–dorsal raphe (DR) circuit is involved in the response to challenging situations, such as during a stress-related task [20].

The vACC is deeply involved in the pathophysiology and treatment of stress-related disorders [8,9,21,22]. Hence, an abnormally increased activity of the vACC has been reported in patients with major depressive disorder (MDD) [23,24], and deep brain stimulation (DBS) of the vACC results in clinical improvements in treatment-resistant MDD patients [25,26,27]. Likewise, a recent neuroimaging study found that MDD patients had higher task-induced vACC hyperactivity than controls, which normalized after a single dose of the fast-acting antidepressant ketamine [28].

Despite the large differences in complexity between human and rodent brains in terms of connectivity and function, the infralimbic (IL) and prelimbic (PrL) cortices are considered to be the rodent homologues of the human vACC and dorsolateral PFC, respectively [8,10,13,29]. In particular, the vACC and IL are deeply involved in fear extinction and in antidepressant actions [8,30]. Hence, the optogenetic activation of IL in rats leads to immediate and enduring antidepressant effects, similar to ketamine [31]. Furthermore, the local application in the IL of ketamine or the selective serotonin reuptake inhibitor (SSRI) citalopram mimics the antidepressant-like effects of their systemic administration [31,32]. A dense population of Layer V pyramidal neurons in the IL and PrL project to the DR [10]. Although anatomical evidence indicates that the PrL projects to the DR more densely than the IL [31], functional data suggest that the IL exerts a more marked control of DR activity than the PrL [32,33]. Hence, an acute increase in glutamatergic neurotransmission in rat IL induced by the blockade of the astroglial glutamate transporter, GLT-1, triggered immediate antidepressant-like effects, which were temporally associated to an enhancement of 5-HT release [32]. Both effects were cancelled by the prior inhibition of 5-HT synthesis, suggesting the involvement of the 5-HT system in behavioral effects. Interestingly, no effects—neurochemical or behavioral—occurred when GLT-1 blockades were performed in the PrL, despite evoking a comparable augmentation of extracellular glutamate than in the IL [33].

On the other hand, a sustained increase in glutamatergic activity in the IL induced by small interfering RNA (siRNA) strategies towards the astroglial glutamate transporters GLAST and GLT-1 [34] triggered a depressive-like phenotype in mice, which was reversed by citalopram and ketamine [34]. These behavioral alterations were accompanied by decreased 5-HT function and reduced hippocampal BDNF expression. Again, the application of siRNAs in the PrL, despite reducing GLAST and GLT-1 expression, as occurred in the IL, did not induce depressive states nor reduced 5-HT function [34].

Overall, these observations add further support to the relevance of the IL (and of the vACC in humans) in mood control and strongly suggest a differential modulation of serotonergic activity by the IL and PrL.

Given the different role of the IL and PrL in terms of their antidepressant effects, depressive-like behaviors, and their associated changes in serotonergic function, here we examined the control exerted by the IL and PrL cortices on serotonergic function in the DR under the working hypothesis that each area may distinctly modulate 5-HT neuronal activity, possibly via differential excitatory inputs onto 5-HT and GABA neurons within the DR. To this end, we assessed the effects of electrical stimulation of the IL and PrL on 5-HT neuronal activity as well as the effects of electrical and optogenetic stimulation of the IL and PrL on 5-HT release in the DR, taken as an overall measure of neuronal activity.

## 2. Results

### 2.1. Modulation of 5-HT Neuronal Responses by Low Frequency Electrical Stimulation of IL and PrL

**Figure 1:**
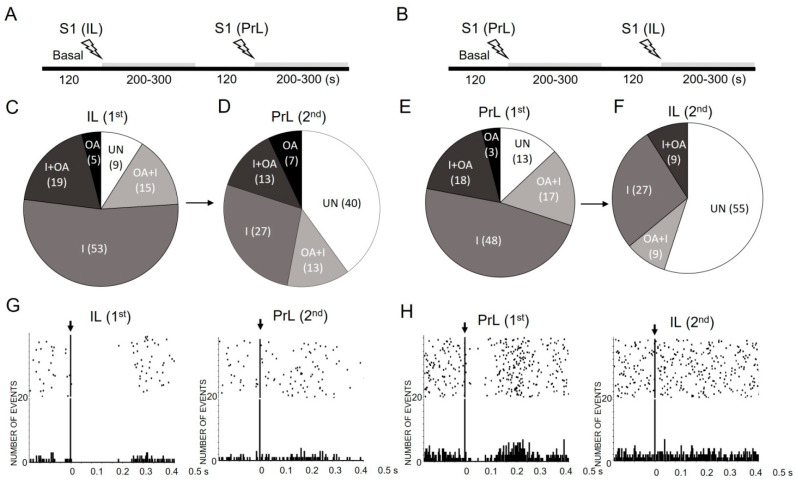
Effects of low-frequency electrical stimulation of infralimbic (IL) and prelimbic (PrL) cortices on 5-HT neuronal responses in dorsal raphe (DR). (**A**,**B**) Schematic representation of the procedures used to examine the responses of DR 5-HT neurons to IL→PrL (**A**) and PrL→IL (**B**) stimulation at a low frequency (S1; 0.9 Hz, 0.2 ms, 1.7 mA). Two different stimulation electrodes were placed in the IL and PrL subdivisions of the same rat (same hemisphere). After 120 s of stable recording (basal), one of the electrodes (IL or PrL) was stimulated, and the response evoked in an identified 5-HT neuron was recorded for 200–300 s. After a 120 s period, we examined the response of the same 5-HT neuron to the stimulation of the electrode placed in the other subdivision (PrL or IL) using exactly the same stimulation characteristics (0.9 Hz, 0.2 ms, 1.7 mA; 200–300 s recordings). (**C**–**F**) Pie charts showing the proportions (%) of the different response types evoked in DR 5-HT neurons by IL and PrL stimulation at a low frequency (S1, 0.9 Hz). Abbreviations: pure inhibitory response (I); pure orthodromic activation (OA); orthodromic activation followed by inhibition (OA + I); inhibition followed by orthodromic activation (I + OA); unaffected (UN). Figures (**C**,**D**) represent the responses of 5-HT neurons first to IL (1st) and then to PrL (2nd) stimulations, whereas figures (**E**,**F**) show the responses of 5-HT neurons first to PrL (1st) and then to IL (2nd) stimulations. Figures (**G**,**H**) show representative peristimulus—time histograms (PSTHs) for IL→PrL ((**G**), pure inhibition→unaffected) and for PrL→IL ((**H**), inhibition followed by activation→unaffected) driven stimulations. Note (1) the predominance of inhibitory (I) responses to both IL and PrL stimulation (53% vs. 48%, respectively) (pie charts (**C**) vs. (**E**), chi-square test n.s.), and that (2) the second stimulation resulted in a very large percentage of unresponsive neurons (40% to IL; 55 to PrL) (pie charts (**C**) vs. (**F**), chi-square test *p* < 0.0001; pie charts (**D**) vs. (**E**), chi-square test *p* < 0.0001; pie charts (**D**) vs. (**F**), chi-square test *p* < 0.01).

First, we examined the effect of IL and PrL electrical stimulation at a low, resting frequency (S1; 0.9 Hz, 0.2 ms, 1.7 mA) on DR 5-HT neurons. We recorded the responses of 47 5-HT neurons to IL stimulation (first stimulation) of which 15 were subjected to a second (PrL) stimulation (Figure 1A, IL→PrL). Likewise, we recorded the responses of 54 neurons to PrL stimulation, of which 11 were subjected to a second (IL) stimulation (Figure 1B, PrL→IL). The basal firing rates of the recorded neurons were 1.36 ± 0.10 and 1.26 ± 0.09 spikes/s for the IL (*n* = 47) and PrL (*n* = 54) stimulations, respectively.

Supplemental Figure S1 shows representative examples of the different response types evoked by the IL and PrL stimulation. Most of the DR 5-HT neurons responded to the IL or PrL stimulation (91% to IL, 87% to PrL) with four different types of responses: inhibition (I) (Supplemental Figure S1A), orthodromic activation (OA) (Supplemental Figure S1B), (activation followed by inhibition) (OA + I) (Supplemental Figure S1C) and inhibition followed by activation (I + OA) (Supplemental Figure S1D).

Nearly half of the evoked responses were purely inhibitory (53% to IL; 48% to PrL), which was followed by I + OA (19% to IL; 18% to PrL), OA + I (15% to IL; 17% to PrL), and a minority of pure OA (5% to IL; 3% to PrL) (Figure 1C,E). The chi-square test revealed no significant differences in the proportion of the responses evoked in the DR 5-HT neurons by the IL (Figure 1C) or PrL (Figure 1E) stimulation.

However, and much to our surprise, the second stimulation site, either PrL (Figure 1D) or IL (Figure 1F)—using exactly the same stimulation protocol, as above—evoked an entirely different pattern of response. From a total of 26 DR 5-HT neurons (15 stimulated from PrL→IL, 11 from IL→PrL), 21 neurons responded differently to the second stimulation while only 5 neurons maintained the same response type. Hence, the second stimulation resulted in a very large percentage of unresponsive neurons (40% of those stimulated first in the IL and then in the PrL, i.e., IL→PrL, and 55% of those subjected to the sequential PrL→IL stimulation). Figure 1G,H show representative examples of neurons responding to the first but not to the second stimulation, either IL→PrL or PrL→IL. The chi-square test revealed the existence of significant differences between the proportion of the different responses in the IL or PrL during the first and second stimulation (pie charts in Figure 1C vs. Figure 1F, *p* < 0.0001 and Figure 1D vs. Figure 1E, *p* < 0.0001), together with differences between the response patterns evoked by the second stimulation in the IL and PrL (Figure 1D vs. Figure 1F, *p* < 0.01).

To avoid an artifactual origin of the observed differences between the first and second stimulations, we examined the response of the same DR 5-HT neurons to a second stimulation from the same site using exactly the same lengths of time and experimental protocols without changing the stimulation site (i.e., IL→IL, PrL→PrL; *n* = 7 in each site). All the neurons examined maintained the response type during the second stimulation from the same site (IL: 5 I, 1 OA + I, 1 UN; PrL: 6 I, 1 OA + I) (Supplemental Figure S2).

### 2.2. Modulation of 5-HT Neuronal Discharge by Electrical Stimulation of IL and PrL

**Figure 2:**
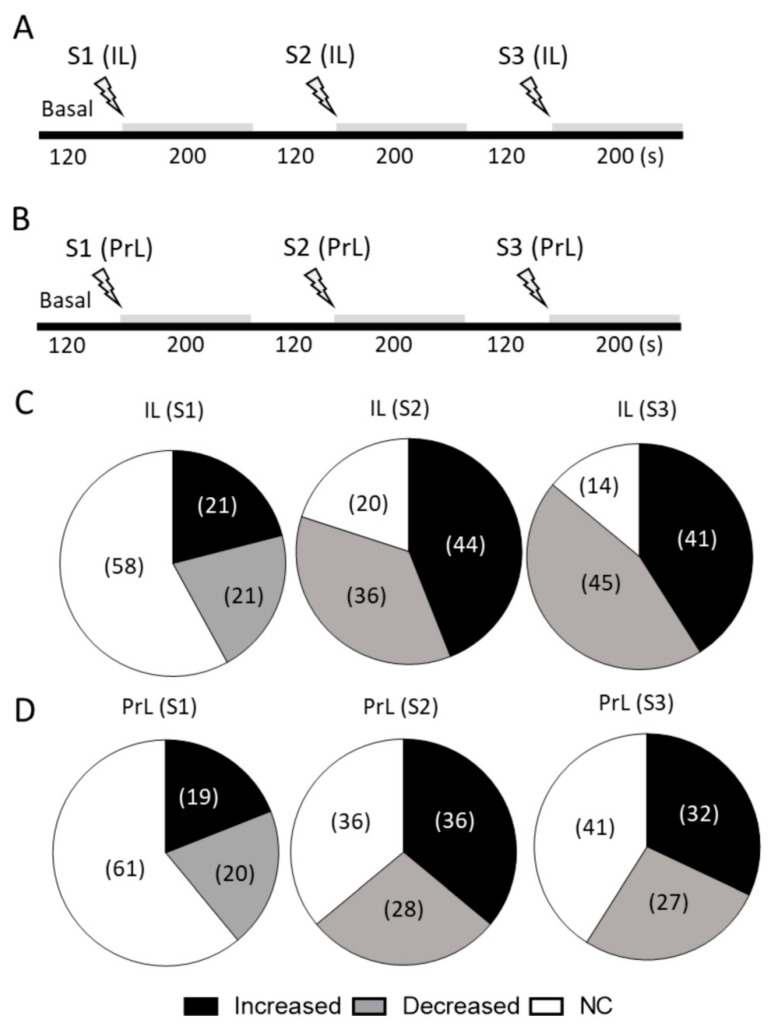
Effects of electrical stimulation at increasing frequencies of infralimbic (IL) and prelimbic (PrL) neurons on 5-HT neuronal discharge in dorsal raphe (DR). (**A**,**B**) Schematic representation of the procedures used to examine the responses of DR 5-HT neurons to IL (**A**) and PrL (**B**) stimulation at increasing frequencies (S1: 0.9 Hz, 0.2 ms, 1.7 mA; S2: 10 Hz, 1 ms, 0.5 mA; S3: 20 Hz, 1 ms, 0.5 mA; 200 s for each stimulation, 120 s between each). In this case, one stimulation electrode was placed in either the IL or PrL subdivisions of the same rat (same hemisphere). (**C**,**D**) Pie charts showing the proportions (%) of the different effects (increasing, decreasing, and no change (NC)) evoked in DR 5-HT neurons by IL (**C**) and PrL (**D**) stimulation under S1, S2 and S3 conditions. Note: (1) the similar proportion of neurons that did not change their firing rate during both IL and PrL stimulation under S1 conditions (61% vs. 58%, respectively) (pie charts IL (S1) vs. PrL (S1), chi-square test n.s.); (2) the higher percentage of serotonergic neurons that changed their firing rate during IL compared to PrL stimulation under S2 and S3 conditions (80% vs. 64% at 10 Hz; 89% vs. 59% at 20 Hz, respectively) (chi-square test IL (S2) vs. PrL (S2), *p* < 0.05); (chi-square test IL (S3) vs. PrL (S3), *p* < 0.0001).

Next, we examined the effect of IL or PrL stimulation at increasing frequencies on the firing rate of DR 5-HT neurons. We recorded 101 serotonergic neurons, 47 being subjected to IL and 54 to PrL stimulation, at increasing frequencies (S1: 0.9 Hz, 0.2 ms, 1.7 mA; S2: 10 Hz, 1 ms, 0.5 mA; S3: 20 Hz, 1 ms, 0.5 mA; 200 s for each stimulation, 120 s between each) (Figure 2A,B). Higher stimulation frequencies (10 and 20 Hz) are representative of the phasic activity of cortical neurons and may mimic the effects of an enhanced glutamatergic input under the examined experimental conditions [35,36]. As stated in Methods section, the stimulations at 10 and 20 Hz were conducted at a lower intensity (0.5 mA), as has been previously used in our laboratory, to prevent damaging the cortical tissue [11]. All serotonergic cells (101) were stimulated under the S1 conditions, and of them 50 were stimulated under the S2 conditions, of which 44 were stimulated under the S3 conditions.

When considering the individual changes from baseline, the IL or PrL stimulation at low frequency (0.9 Hz, S1) evoked similar changes in terms of 5-HT neuronal discharge (Figure 2C,D) (chi-square test, n.s.). However, the electrical stimulation of the IL and PrL at higher frequencies differently modulated the mean firing rate of serotonergic neurons. Hence, the proportion of increased and decreased 5-HT neurons was greater after the IL stimulation than after the PrL stimulation: 80% (IL) vs. 64% (PrL) under S2 conditions (chi-square test IL vs. PrL at 10 Hz, *p* < 0.05) and 86% (IL) vs. 59% (PrL) under S3 conditions (chi-square test IL vs. PrL at 20 Hz, *p* < 0.0001) (Figure 2C,D).

**Figure 3:**
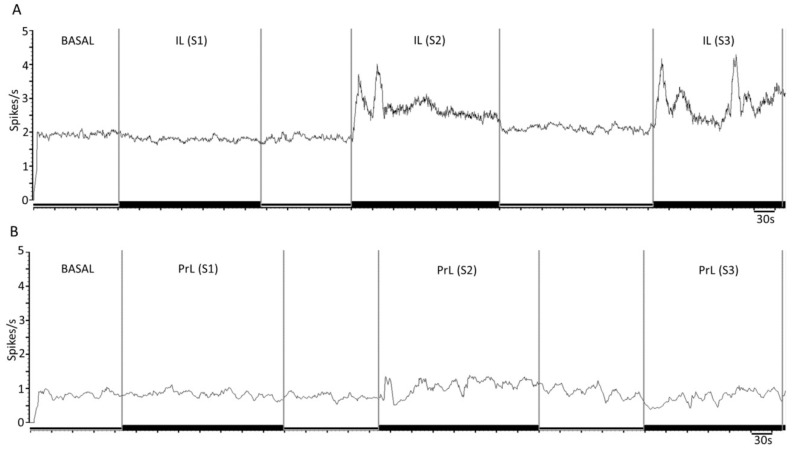
Representative example of the effect of electrical stimulation at increasing frequencies of infralimbic (IL) and prelimbic (PrL) neurons on 5-HT neuronal discharge in dorsal raphe (DR). (**A**,**B**) Mean frequency graphs showing spikes/s of representative recordings of DR 5-HT neurons during IL (**A**) and PrL (**B**) electrical stimulation at increasing frequencies (S1: 0.9 Hz, 0.2 ms, 1.7 mA; S2: 10 Hz, 1 ms, 0.5 mA; S3: 20 Hz, 1 ms, 0.5 mA; at least 200 s for each stimulation, 120 s between each). Note that IL (but not PrL) stimulation modulated the neuronal discharge of the recorded neurons (**A**) (no change→increasing→increasing for S1→S2→S3, respectively).

Figure 3 shows representative recordings of the DR 5-HT neurons after the IL (Figure 3A) or PrL (Figure 3B) electrical stimulation at increasing frequencies.

### 2.3. Pharmacological Characterization of IL- and PrL-Induced Responses in DR 5-HT Neurons: Involvement of 5-HT_1A_ and GABA_A_ Receptors

**Figure 4:**
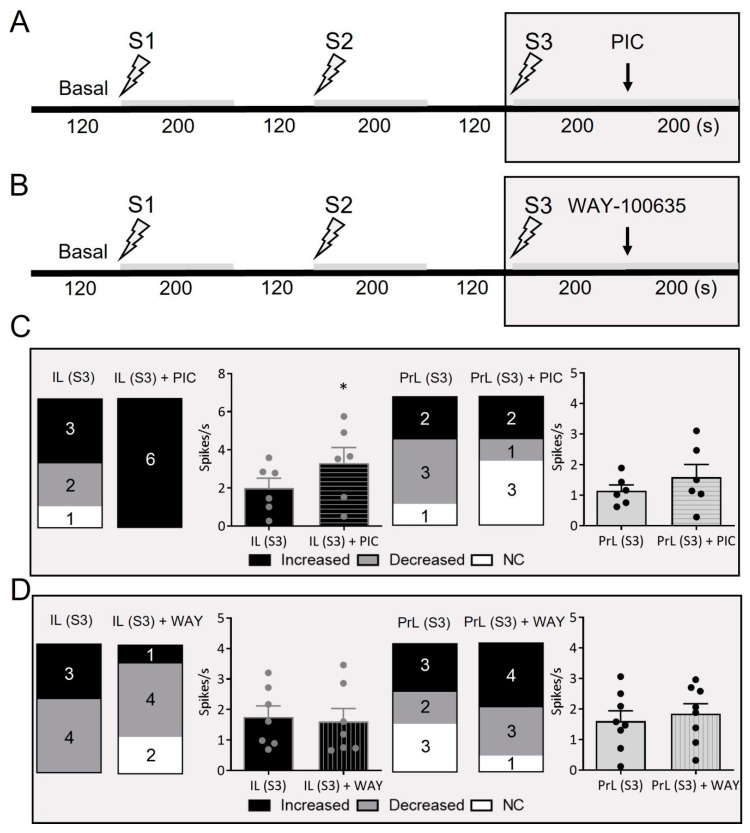
Involvement of 5-HT_1A_ and GABA_A_ receptors in IL and PrL induced responses in DR 5-HT neurons. (**A**,**B**) Protocol used to examine the responses of DR 5-HT neurons to IL and PrL stimulation at increasing frequencies (S1: 0.9 Hz, 0.2 ms, 1.7 mA; S2: 10 Hz, 1 ms, 0.5 mA; S3: 20 Hz, 1 ms, 0.5 mA) before and during picrotoxinin (PIC, GABA_A_-R antagonist, 2 mg/Kg i.v.) (**A**) and WAY-100635 (5-HT_1A_-R antagonist,10 µg/Kg i.v.) (**B**) administration. Note that both drugs were injected after S3, and the stimulation was maintained for at least 200 s more, to evaluate the effects of the drug. (**C**,**D**) Picrotoxinin (PIC) (**C**) and WAY-100635 (**D**) effects on IL- and PrL-induced responses under S3 conditions. Bars show the number of increased, decreased, and unaffected (NC) 5-HT neurons before and after PIC and WAY administration during S3 stimulation. Bar graphs indicate firing rate (spikes/s) for each stimulation site (IL and PrL) and drug (PIC and WAY-100635). Note that the GABA_A_ receptor antagonist PIC (but not WAY-100635, a 5-HT_1A_ receptor blocker) totally changed the effects on serotonergic neuron activity induced by IL stimulation under S3 conditions, increasing their firing discharge (* *p* < 0.05 vs. IL (S3)).

The mPFC regulates serotonergic activity in the DR by direct excitatory inputs from layer-V pyramidal neurons. Excitations are mediated by the activation of AMPA-R and NMDA-R on 5-HT neurons, whereas inhibitions are due to (i) local negative feedback through 5-HT_1A_ autoreceptors and (ii) the activation of local GABA interneurons and the subsequent inhibition of 5-HT neurons via GABA_A_-R [11,37]. The latter mechanism also appears to involve activation by 5-HT of5-HT-Rs expressed in local GABA neurons, such as 5-HT_1A_-R [38], 5-HT_2B_-R [39], or 5-HT_2C_-R [40]. We therefore examined the involvement of GABA_A_-R and 5-HT_1A_-R in IL- and PrL-evoked responses at 20 Hz (S3) by administering the respective antagonists picrotoxinin (PIC, 2 mg/Kg) (Figure 4A) and WAY-100635 (10 µg/Kg) (Figure 4B).

The effect of systemic picrotoxinin administration on serotonergic discharge during electrical stimulation under S3 conditions was examined in 12 rats, 6 stimulated from the PrL and 6 from the IL, whereas WAY-100635 was injected to 15 rats, 8 stimulated from the PrL and 7 from the IL. Figure 4C,D show the proportion of 5-HT neurons with increased, decreased, and unaffected discharge rates before and after drug administrations. Remarkably, the GABA_A_ receptor antagonist PIC (but not WAY-100635) enhanced the discharge rate of all the 5-HT neurons subjected to IL stimulation under S3 conditions, (from 2.0 ± 0.5 to 3.3 ± 0.8 spikes/s; *p* < 0.05) (Figure 4C,D). In contrast, PIC reversed the inhibitory effect of PrL stimulation under S3 conditions in two units, but failed to significantly increase the discharge rate (Figure 4C). Likewise, as was also observed in the IL-stimulated 5-HT neurons, WAY-100635 failed to significantly change the 5-HT neuron discharge in the PrL-stimulated group (Figure 4D).

**Figure 5:**
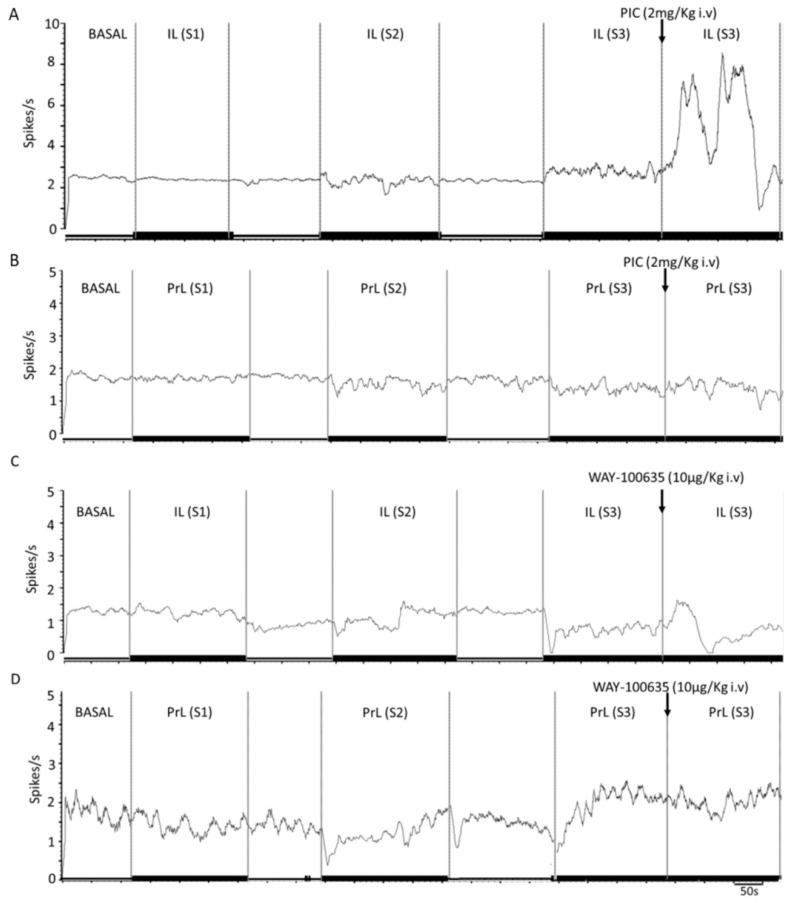
Involvement of 5-HT_1A_ and GABA_A_ receptors in IL- and PrL-induced responses in DR 5-HT neurons. (**A**–**D**) Mean frequency graphs showing spikes/s of representative recordings of DR 5-HT neurons during IL and PrL stimulation at increasing frequencies (S1: 0.9 Hz, 0.2 ms, 1.7 mA; S2: 10 Hz, 1 ms, 0.5 mA; S3: 20 Hz, 1 ms, 0.5 mA) and pharmacological effects of picrotoxinin (PIC, GABA_A_-R antagonist) (**A**,**B**) and WAY-100635 (5-HT_1A_-R antagonist) (**C**,**D**) during S3 stimulation. Note that PIC (but not WAY-100635) increased serotonergic discharge during IL stimulation above values under S3 conditions (**A**).

Representative recordings of the effects of PIC and WAY-100635 in IL- and PrL-stimulated 5-HT neurons are shown in Figure 5.

### 2.4. Modulation of 5-HT Release in DR by Electrical Stimulation of IL and PrL

**Figure 6:**
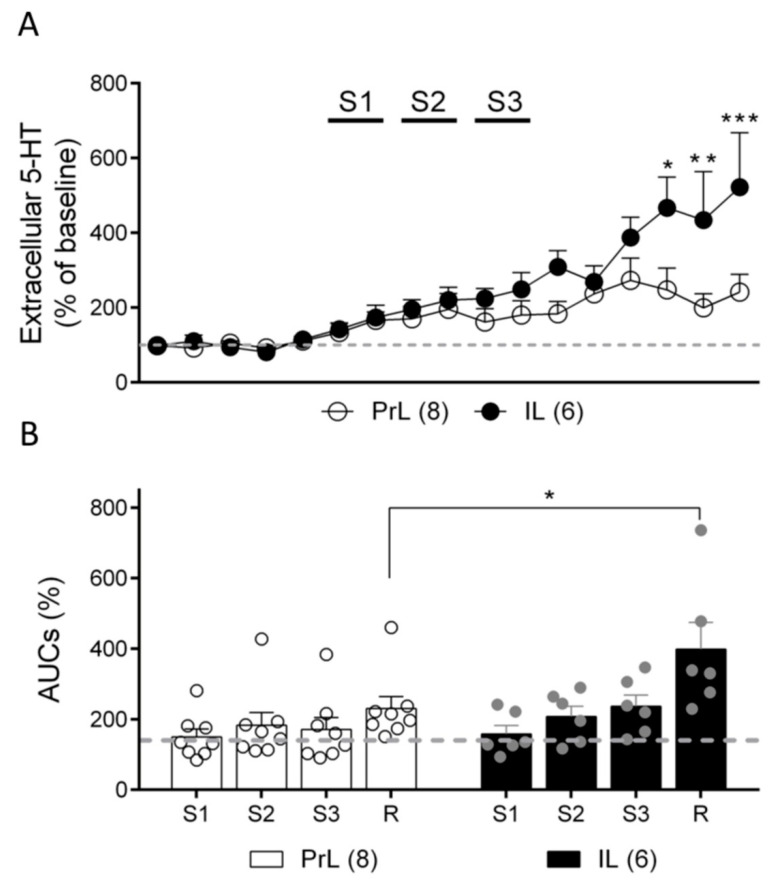
Effect of IL and PrL electrical stimulation (S1: 0.9 Hz, 0.2 ms, 1.7 mA; S2: 10 Hz, 1 ms, 0.5 mA; S3: 20 Hz, 1 ms, 0.5 mA) on extracellular 5-HT concentration in DR. (**A**) Graph shows data as % of basal values averaged from five pre-stimulation samples. Note that both electrical stimulations of IL and PrL enhanced 5-HT release in the DR in a frequency-dependent manner. More marked effects were found 30–60 min after the end of IL stimulation. (**B**) Bar graph showing that similar results were found when data were treated as normalized areas under curve (AUC) corresponding to each stimulation (S1–S3) and recuperation (R, samples 12–17) periods. * *p* < 0.05, ** *p* < 0.01, *** *p* < 0.001 vs. PrL.

Next, we assessed the effect of IL or PrL electrical stimulation on extracellular 5-HT concentrations in the DR. This variable was used as a surrogate measure of the overall 5-HT neuron activity in the area, as sampled by microdialysis probes. The anesthesia conditions and stimulation settings were the same as those used in the electrophysiological experiments. The DR extracellular 5-HT concentrations at basal conditions were 8.5 ± 0.5 fmol/fraction (*n* = 14). The electrical stimulation of the IL enhanced the 5-HT release in the DR in a frequency-dependent manner to a maximum of 237 ± 32% of that of the baseline at 20 Hz (S3), while the stimulation of the PrL elevated the 5-HT release to 183 ± 37% of that of the baseline at a frequency of 10 Hz (S2). Sixty minutes after the end of the stimulation, the extracellular 5-HT concentration was further enhanced to 522 ± 145% of that of the baseline in the IL and 242 ± 47% of that of the baseline in the PrL (Figure 6A,B) (two-way ANOVA: stimulation effect: F (16, 192) = 13.17, *p* < 0.0001; area effect: F (1, 12) = 3.784, n.s.; stimulation x area interaction: F (16, 192) = 3.036; *p* < 0.001) (Figure 6A). Similar results were obtained when the AUCs (%) for each stimulation were considered (two-way ANOVA; stimulation effect: F (3, 36) = 11.67, *p* < 0.0001; area effect: F (1, 12) = 2.609, n.s.; stimulation x area interaction: F (3, 36) = 3.187, *p* < 0.05) (Figure 6B). In both cases, post-hoc differences between the IL and PrL were found after ending the S3 stimulation.

### 2.5. Modulation of 5-HT Release in DR by Optogenetic Stimulation of IL and PrL

**Figure 7:**
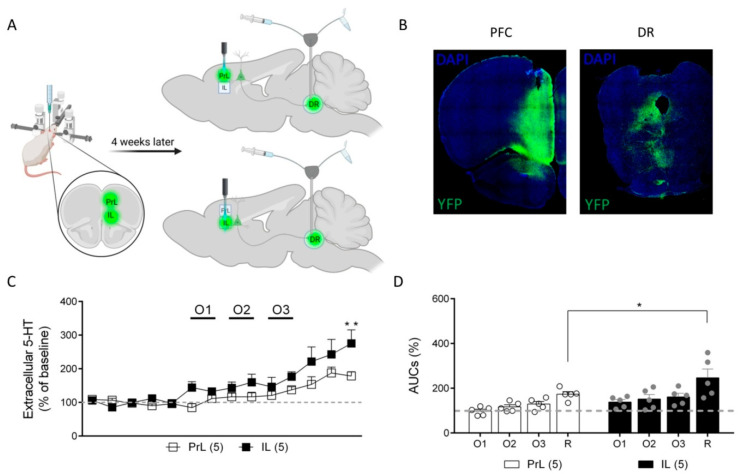
Effect of IL and PrL optogenetic stimulation (O1: 1 Hz, O2: 10 Hz, O3: 20 Hz, 5 ms pulse width, and ~5 mW at the fiberoptic tip) on extracellular 5-HT concentration in DR. (**A**) Schematic representation of the experimental protocol used. (**B**) Representative fluorescent image of a coronal slice of a rat brain at the level of AAV injection site (mPFC, left panel) and at the microdialysis probe implant site (DR, right panel). Note the intense fluorescence in DR, indicating a successful expression ofAAV-CHR2-YFP by mPFC-DR descending axons. (**C**) Graph showing data as % of basal 5-HT values averaged from five pre-stimulation samples. Note that optogenetic stimulation of both IL and PrL enhanced 5-HT release in the DR in a frequency-dependent manner. More marked effects were found 30 min after the end of IL stimulation. (**D**) Bar graph showing that similar results were found when data were analyzed as normalized areas under curve (AUC) corresponding to each stimulation (O1–O3) and recuperation (R, samples 12–14) periods. * *p* < 0.05; ** *p* < 0.01 vs. PrL.

We next used an optogenetic approach to selectively stimulate cortical excitatory neurons in either the IL or PrL cortices and evaluate their effects on serotonin levels in the DR. To this end, we injected an AAV construct that expressed ChR2 under a CamKII promoter in the mPFC, and 4 weeks later we placed a microdialysis probe in the DR and a fiberoptic cannula in either the PrL or IL cortices in anesthetized rats (Figure 7A). Figure 7B shows a representative coronal section of a rat brain at the level of the AAV injection site (mPFC) and at the microdialysis probe implant site (DR). Note the intense fluorescence in the DR, indicating a successful expression of AAV-CamKII-ChR2-YFP constructs by mPFC and along their descending axons in the DR.

In this set of experiments, the basal 5-HT extracellular concentrations in DR were 8.0 ± 1.3 fmol/fraction (*n* = 10). The optogenetic stimulation of the IL neurons increased the extracellular 5-HT concentration to a maximum of 161 ± 16% of that of the baseline in the DR at the O3 (20 Hz) stimulation frequency, while the stimulation of the PrL neurons at the same frequency elevated 5-HT to 129 ± 11% of that of the baseline. Thirty minutes after ending the optogenetic stimulations, the extracellular 5-HT concentration in the DR was further enhanced to 247 ± 39% of that of the baseline in the IL and to 173 ± 12% of that of the baseline in the PrL (Figure 7C,D) (two-way ANOVA: stimulation effect: F (13, 104) = 13.53, *p* < 0.0001; area effect: F (1, 8) = 5.388, *p* < 0.05; stimulation x area interaction: F (13, 104) = 1.803, *p* = 0.052) (Figure 7C). Considering the AUCs (%) for each stimulation period, significant differences were also found (two-way ANOVA; stimulation effect: F (3, 24) = 14.86, *p* < 0.0001; area effect: F (1, 8) = 5.723, *p* < 0.05; stimulation x area interaction: F (3, 24) = 0.8379, n.s.) (Figure 7D). In both cases, the post-hoc differences between the IL and PrL 30 min after ending the O3 stimulation were found.

## 3. Discussion

In the present study, we investigated whether infralimbic (IL) and prelimbic (PrL) inputs to the DR elicited differential responses in 5-HT neurons, as suggested by the distinct role of both the mPFC subdivisions in emotional control (see the Introduction). This study was also prompted by previous data from our lab indicating that acute stimulation of AMPA-R in the IL (but not in the PrL) evoked an immediate antidepressant-like response in rats, which was associated with an enhancement of 5-HT release [32,33]. The present electrophysiological observations indicated that electrical stimulation of both the IL and PrL at a low frequency (0.9 Hz) induced a comparable overall inhibitory effect on the DR 5-HT neurons. Regional differences were found when the IL and PrL were stimulated at higher frequencies, which was representative of phasic mPFC activity, with a higher percentage of serotonergic neurons changing their mean firing rate to IL compared to PrL stimulation (80% vs. 64% at 10 Hz; 86% vs. 59% at 20 Hz, respectively). The responses of the 5-HT neurons at 20 Hz IL stimulation were sensitive to GABA_A_ (but not 5-HT_1A_) receptor blockade, suggesting the involvement of local GABA_A_-R inputs in the control of DR 5-HT neuron activity by IL inputs. We also showed that the electrical and optogenetic stimulation of both the IL and PrL enhanced 5-HT release in the DR, with a greater effect of IL compared to PrL stimulation. To our knowledge, no previous study has examined the potential differential control of serotonergic activity by the IL and PrL. Given the key role of the PFC and of the 5-HT system in MDD, the present observations may help to better understand the brain circuits involved the pathophysiology and treatment of this psychiatric disorder.

Interestingly, a very large proportion of the DR 5-HT neurons were sensitive to both stimulation sites (91% to IL, 87% to PrL) (Figure 1C,E), indicating that both the IL and PrL are key areas in the control of serotonergic activity. This large proportion was consistent with the existence of a large number of DR-projecting layer-V pyramidal neurons in both the mPFC subdivisions [14], yet with distinct trajectories to the DR [13]. Previous electrophysiological evidence has revealed that mPFC pyramidal neurons might activate or inhibit DR 5-HT neurons [11]. These types of excitations are monosynaptic and involve the activation of the AMPA and *N*-methyl-D-aspartate (NMDA) receptors on serotonin neurons [11,41], whereas these types of inhibitions are mediated by two different mechanisms, namely (i) a local negative feedback recruiting 5-HT_1A_ autoreceptors and (ii) the activation of GABA interneurons in the DR by mPFC inputs and the subsequent inhibition of 5-HT neurons [11,37,42,43]. Consistent with these previous studies, we found that both IL and PrL stimulation at a low resting frequency (0.9 Hz) inhibited a similar proportion of DR 5-HT neurons (53% to IL, 48% to PrL).

In our first set of experiments, the same 5-HT neuron was stimulated twice (IL-to-PrL and PrL-to-IL, same hemisphere) with a 2 min period between each manipulation. Surprisingly, the second stimulation (either IL or PrL) induced a completely different pattern of response, leaving almost half of the recorded neurons insensitive to the second stimulation. This differential response to the second stimulation site did not appear to be an experimental artifact, since the response was maintained after a second stimulation from the same site (either IL or PrL). Although we do not have a clear explanation for this observation, it may depend on the intrinsic circuitry between the IL and PrL. Anatomical [44] and electrophysiological [45,46] evidence suggest a significant interaction between the IL and PrL. Hence, the IL and PrL exhibited reciprocal layer V/VI connectivity [47,48,49], and the optical activation of the IL pyramidal neurons inhibited the PrL pyramidal cells, indicating that the IL controls the PrL output [45]. Disconnection of the IL and PrL disrupted the neural rhythms within the mPFC [46], and an increased interregional synchrony between the IL and PrL was found during fear extinction [49]. Taken together, these data suggest a complex circuit-based system within the PFC that may affect the activity of layer-V neurons projecting to the DR. However, the elucidation of the precise circuit mechanisms requires further research.

A key finding of the present study was that the IL and PrL exerted a differential control of DR 5-HT activity when stimulated at higher frequencies (10–20 Hz), which was representative of the phasic activity of the mPFC and likely mimicked the effects of an enhanced excitatory neurotransmission. Despite evoking transient effects at the initial stages of stimulation (see Figure 3 and Figure 5), the IL stimulation at 10 and 20 Hz induced an overall change in the activity of DR 5-HT neurons that was more marked compared to the PrL stimulation at the same frequencies (80% vs. 64% at 10 Hz; 86% vs. 59% at 20 Hz, respectively). Although the PrL shows denser projections to the DR compared to the IL [13], our results were in accordance with the more prominent role of the IL vs. PrL in regulating the antidepressant-like actions associated with the modulation of 5-HT activity [32,33]

Interestingly, the mechanisms involved in the stimulation of DR 5-HT neurons by the IL and PrL appeared to differ, with a more marked involvement of the GABA neurons in the DR in the case of the IL, as suggested by their sensitivity to the GABA_A_-R antagonist picrotoxinin. Hence, in addition to direct excitatory inputs in 5-HT neurons [11], DR-projecting pyramidal neurons in the IL would preferentially (compared to PrL pyramidal neurons) target GABAergic interneurons in the DR [11,37]. This view is also consistent with previous reports demonstrating how high-frequency stimulation (HFS) of the rodent IL part of the PFC induces a preferential inhibitory effect on DR neurons mediated by GABA_A_ receptors [43,50]. Likewise, the intra-DR administration of GABA antagonists enhanced 5-HT cell firing and release [51,52].

However, the 5-HT_1A_-R antagonist WAY-100635 was unable to augment the effect of IL and PrL stimulation on DR 5-HT activity. This result was totally unexpected, given the marked role of the self-inhibitory mechanisms mediated by 5-HT_1A_ autoreceptors in the control of DR 5-HT neuronal activity and 5-HT release [11,53,54]. Hence, low-frequency stimulation of mPFC inhibited a large proportion of DR 5-HT neurons via 5-HT_1A_ autoreceptor activation [11]. The present results may indicate that stimulation at higher rates would overcome the self-inhibitory mechanisms mediated by 5-HT_1A_ autoreceptors by increasing the excitation/inhibition ratio within the DR.

The differential role of the IL and PrL in the control of DR 5-HT activity was further supported by the in vivo microdialysis studies during the electrical and optical stimulation of the IL and PrL. We showed that the stimulation of both areas enhanced 5-HT release in the DR. Similar results were obtained in a previous study from our group, showing that the electrical stimulation of the mPFC enhanced DR 5-HT release [11]. Here, we also used optogenetic techniques to provide an additional degree of selectivity in the study of the mPFC-DR pathway. We found that the viral-mediated expression of ChR2 in the excitatory pyramidal neurons of the IL and PrL induced a robust ChR2 expression in the terminal fields within the DR (see Figure 7B). This observation confirmed the correct AAV infection site and further supports the idea that the IL and PrL are key areas in the control of serotonergic activity, with a significantly greater enhancement of DR 5-HT release after IL stimulation.

This finding adds to previous data, confirming a relevant role for the IL in mood disorders and antidepressant treatment [19,31,33,55,56,57,58]. In particular, the present data agreed with previous data from our laboratory, which showed that AMPA-R activation in the IL (but not in the PrL) evoked fast and robust antidepressant-like effects in rats, depending on the activation of the descending excitatory pathways to the DR and the subsequent enhancement of serotonergic neurotransmission [32,33].

It is noteworthy that the increase in 5-HT release in the DR induced by both electrical and optogenetic stimulations persisted after the cessation of the stimulation and increased over time. Although the mechanisms involved in this long-term potentiation (LTP-)-like effect remain to be elucidated, they were consistent with the modulation of 5-HT neuron activity by NMDA receptors [7] and were similar to those found when recording DR 5-HT neurons before, during, and after IL high-frequency stimulation [59]. Interestingly, Haj-Dahmane and colleagues (2017) reported on a novel mechanism for the spike-timing-dependent LTP of glutamate synapses in rat DR 5-HT neurons [60].

We found that 5-HT release in the DR was greater during the electrical compared to the optical stimulation of the IL and PrL. The electrical stimulation non-specifically activated multiple types of neurons near the implant site, whereas the optogenetic manipulation allowed the direct and selective activation of pyramidal neurons in a specific-cell manner. The observed differences in terms of the extent of 5-HT release may also reflect the distinct effect at the local level (i.e., the stimulation site) as well as at the circuit level, given that the stimulation of pyramidal neurons in the IL and PrL could regulate the activity of the other brain regions (e.g., lateral hypothalamus, ventral tegmental area, locus coeruleus, etc.) involved, and in turn, in the modulation of 5-HT neuronal activity [10].

The main limitation of the present study was the use of only male naïve anesthetized rats. However, the use of chloral-hydrate-anesthetized male naïve rats allowed a direct comparison with previous studies [11,42] examining the action of PFC stimulation on DR 5-HT activity. An extension of the present observations to female rats and rodent MDD models would be highly desirable. An additional experimental limitation was the use of rats that expressed ChR2 in the entire mPFC for the selective optogenetic stimulation of the IL and PrL, thus potentially affecting the local circuitry between both areas [44,45,46,47,48,49].

Overall, the present study highlights the differential roles for the IL and PrL in modulating serotonergic activity and neurotransmitter release in the DR. More marked differences were found when the stimulations were carried out at higher frequencies, which was representative of the phasic activity of the mPFC and/or stimulation by the glutamatergic agents involved in fast antidepressant actions. Despite its less-dense projections to the DR [13,61], the IL induced a more marked modulation of DR 5-HT activity than the PrL. Given the key role played by 5-HT in MDD treatment and the key role of the vACC (in humans) and IL (in rodent) in MDD and antidepressant strategies, our data might be extremely helpful in clarifying the neural circuitry involved in these processes.

## 4. Materials and Methods

### 4.1. Animals

Male albino Wistar rats weighing 250–350 g were used in this study (Charles River, France). Animals were kept in a controlled environment (12 h light and 12 h dark cycle and a 22  ±  2 °C room temperature), with food and water being provided ad libitum. The experiments were conducted according to the NIH Guide for the Care and Use of Laboratory Animals, Animals (Scientific Procedure) Act 1986, and following the ARRIVE guideline (Kilkenny et al. 2010) during the awake phase. The animal care followed European Union regulations (directive 2010/63 of 22 September 2010) and was approved by the Institutional Animal Care and Use Committees.

### 4.2. Drugs

8-OH-DPAT (5-HT_1A_-R agonist, 30 µg/Kg), WAY-100635 maleate (5-HT_1A_-R antagonist, 10 µg/Kg), and Picrotoxinin (GABA_A_-R antagonist, 2 mg/Kg) were obtained from Sigma/RBI (Natick, MA, USA). 8-OH-DPAT and WAY-100635 were dissolved in saline and stored at −20 °C until use. Picrotoxinin was dissolved in 10% DMSO and prepared on the day of experiments. Doses were expressed as free bases and were chosen according to previous works from our lab and others [11,42,62]. Both compounds were injected intravenously (i.v.) through the femoral vein at a volume of 1 mL/kg.

### 4.3. Stereotaxic Surgery

Stereotaxic coordinates (in mm) were chosen from the bregma and skull (or brain surface) in accordance with the rat atlas of Paxinos and Watson (2005) [63]. All in vivo electrophysiology and microdialysis experiments were carried out in rats under chloral hydrate anesthesia (induction: 400 mg/kg; maintenance: 50–70 mg/kg/h) using a perfusion pump.

For the in vivo electrophysiology, one or two bipolar stimulation electrodes were stereotaxically implanted in each animal in the IL (AP + 3.0, L -0.5, DV -5.4) and/or PrL (AP + 3.5, L -0.7, DV -3.8) (DV from the skull) in the same hemisphere. Serotonergic neurons from DR were recorded at the following coordinates (AP -7.6, L -2.2, DV -5.4 to -7 from the brain surface, with a lateral angle of 20°).

For the in vivo microdialysis, one concentric dialysis probe (Cuprophan; 1.5 mm-long) was fixed in the DR (AP -7.6, L -2.2, DV -7.2 from the skull, with a lateral angle of 20°). The placement of the stimulation electrodes and optic fibers in the IL and PrL were carried out using the same coordinates as in the in vivo electrophysiology.

For the optogenetic stimulation, an adeno-associated virus (AAV) containing Channelrhodopsin (ChR2) under a CaMKII promoter (AAV1-CaMKIIa-hChR2(H134H)-eYFP-WPRE.hGH; AAV-ChR2) was injected under isofluorane anesthesia (5% induction, and 2% maintenance). Meloxicam (2 mg/Kg s.c.) was administrated 30 min before surgery to avoid pain and inflammation. Virus production, amplification, and purification were performed by the University of Pennsylvania -Penn Vector Core (titers: ~1 × 10^12^ genomic particles/mL, Cat #: AV-1-26969P), and a volume of 1 μL of the corresponding viral constructs was injected into the mPFC (0.5 μL in the PrL and 0.5 in the IL, same coordinates as above) by a 5 μL Hamilton syringe with a 33-gauge needle at 0.1 μL/min. The injection needle was left for an additional 5 min period to allow the diffusion of virus particles and to avoid reflux. Four weeks after AAV injection, rats were deeply anesthetized with chloral hydrate, and fiber-optic cannulas (MFC_200/240–0.22_3.5_ZF1.25_FLT; Doric Lenses) were stereotaxically placed in either the IL or PrL, and a microdialysis probe was implanted in the DR as described above.

### 4.4. In Vivo Electrophysiology

Single-unit extracellular recordings of the DR 5-HT neurons in the chloral-hydrate-anaesthetized rats were performed with a glass micropipette filled with 2 M saline as previously described [11,15]. Serotonergic neurons from the DR were recorded during descending tracks and were identified according to previously described electrophysiological criteria [11]. The stimulation parameters were also chosen from [11]. The 5-HT neurons had a regular firing rate with low frequencies (0.3–3 Hz; mean ≈ 1.1 Hz) and were pharmacologically identified by administrating the 5-HT_1A_-R agonist 8-OH-DPAT (30 µg/Kg i.v.) and, subsequently, the 5-HT_1A_-R antagonist WAY-100635 (100 µg/Kg i.v.) (Supplemental Figure S3). Once a spontaneously active 5-HT neuron was found, its discharge was recorded for at least 120 s (basal activity). Then, the IL and/or PrL were stimulated at a low frequency (S1: 0.9 Hz, 1.7 mA, 0.2 ms square pulses) for 200–300 s. In some neurons, the response to higher frequencies and lower-intensity IL or PrL stimulation for 200 s was also assessed using the following settings (S2: 10 Hz, 0.5 mA, 1 ms square pulses; S3: 20 Hz, 0.5 mA, 1 ms square pulses). The time between stimulations was 120 s. The S1 parameters were identical to those used for PTSHs, whereas the stimulations at 10 and 20 Hz were conducted at a lower intensity (0.5 mA) and with 1 ms pulses to mimic the discharge of projection pyramidal neurons and also avoiding the possibility of damaging the cortical tissue [11].

The involvement of 5-HT_1A_-R and GABA_A_-R in the responses induced by IL and PrL stimulation in DR 5-HT neurons was also studied. Thus, either WAY-100635 or picrotoxinin were injected after 200 s of stimulation under S3 conditions, and the stimulation was maintained for at least 200 more seconds.

After the completion of the experimental procedures, the rats were euthanized by an anesthetic overdose, and their brains were rapidly removed to verify the stimulation sites.

#### Electrophysiology Data Analysis

The responses in the DR 5-HT neurons elicited by the IL or PrL electrical stimulation at low frequencies (0.9 Hz, S1) were characterized by measuring the magnitude and duration of the inhibitory and excitatory responses from peristimulus–time histograms (PSTH) (4 ms width) as previously described [11,42]. The following responses were identified: (1) Orthodromic activation (OA): Orthodromic excitations elicited spikes with short and variable latencies and a post-stimulus firing rate that was greater than the mean pre-stimulus firing rate plus 2 times the standard deviation during at least 4 bins [11,42]. The success rate in the orthodromic activations was calculated as the number of spikes during the excitation period divided by the number of triggers. (2) Inhibition: The onset of the inhibition was defined by either a total cessation of spikes for at least four successive bins (4 ms width) or a 75% decrease in the number of spikes with respect to the pre-stimulus value. The offset of the inhibition was defined as the first of four bins equal to or above the pre-stimulus value. The magnitude of the inhibition was calculated as the percentage of firing versus the pre-stimulus (200 ms) firing rate. (3) Orthodromic activation followed by inhibition (OA + I). (4) Inhibition followed by orthodromic activation (I + OA). (5) In addition, some 5-HT neurons did not respond to the IL or PrL stimulation (unaffected, UN). To compare the effects elicited by the IL and PrL electrical stimulation at different frequencies on DR 5-HT neurons, the firing rate (spikes/s) was also calculated before and during (3 min) electrical stimulation. 5-HT neuron activity was considered increased (or decreased) if the firing rate was ≥15% (or ≤15%) of the basal values, respectively.

### 4.5. In Vivo Microdialysis during Electrical or Optogenetic Stimulation

The extracellular 5-HT concentrations in the DR during the electrical or optical stimulation of IL or PrL were measured by in vivo microdialysis as previously described [11,42,62] under chloral hydrate anesthesia. Briefly, a probe placed in the DR was continuously perfused with artificial cerebral spinal fluid (aCSF) at 3.28 μL/min, and 10 min fractions were collected. After six basal samples (without any stimulation), two fractions were collected during IL or PrL electrical or optogenetic stimulation. Once the last stimulation was terminated, either three or six more samples (with no stimulation) were collected. The 5-HT concentrations were analyzed by high-performance liquid chromatography (HPLC) with electrochemical detection (Waters 2465) at +0.75 V with a detection limit of 1–2 fmol/sample.

The 5-HT extracellular concentrations in the DR were expressed as fmol per fraction. The microdialysis results were given as a percentage (%) of the basal values, averaged from 5 pre-stimulation samples and also as normalized areas under curve (AUC %) corresponding to every stimulation period.

#### 4.5.1. Electrical Stimulation

A first group of animals (*n* = 14) were stereotaxically implanted with one stimulating electrode (in the IL or PrL) at the same coordinates used for the electrophysiological studies. The stimulation settings (S1, S2, and S3) were also as described above.

#### 4.5.2. Optogenetic Stimulation

A second group of rats (*n* = 10) received an AAV-ChR2 injection in the IL and PrL. Four weeks later, the rats were deeply anesthetized with chloral hydrate, and fiber-optic cannulas were placed in the IL or PrL, and a microdialysis probe was implanted in the DR. A 473nm blue light was used to stimulate the excitatory neurons expressing ChR2 and was delivered from a diode-pumped solid-state blue laser (Laserglow) at 1 Hz (O1), 10 Hz (O2), and 20 Hz (O3) with a 5 ms pulse width and at ~5 mW (measured at the end of the patchcord) using a custom-made waveform generator (Arduino) as detailed in [11,42,62].

After completion of the experiments, the rats were euthanized by an anesthetic overdose, and their brains were rapidly removed to verify the expression of the AAV construct in the mPFC and the correct placement of the microdialysis probe in the DR. Briefly, the brains were placed in 4% paraformaldehyde, dehydrated in a PBS/sucrose gradient [from 15% (48 h postmortem) to 30% (32 h postmortem)] with 0.02% sodium azide, snap-frozen, and stored at −20 °C.

### 4.6. Immunohistochemistry

Coronal sections (30 µm coronal) were cut on a cryostat Microm HM500M and preserved in PBS with 0.02% sodium azide at 4 °C. Anti-GFP (1:500, Invitrogen, #11122, RRID: AB_221569) antibody was used to evaluate the expression of AAV-ChR2 virus constructs. Free-floating sections were washed in PBS, permeabilized, and blocked for 15 min in PBS containing 0.3% Triton X-100, 0.2% gelatine, and 5% normal donkey serum (S30–100ML Merck Millipore, Burlington, MA, USA). The sections were washed again in PBS and incubated overnight at 4 °C with primary antibodies. The brain slices were washed and incubated for 2 h with a secondary antibody (1:200 donkey anti-rabbit A555 (A-31572. Life Technologies, Carlsbad, CA, USA)) for the GFP visualization only. The sections were washed again in PBS, incubated for 10 min in a Hoescht 33342 1/1000 device (H3570. Life technologies), and mounted on microscope slides using Entellan (Sigma-Aldrich, St. Louis, MO, USA).

#### Image Acquisition and Analysis

Tissue sections were imaged using an inverted Nikon Eclipse Ti2-E microscope (Nikon Instruments, Melville, NY, USA) attached to an Andor Dragonfly 200 spinning disk unit (Oxford Instruments, Abingdon, UK). For all the experiments, a Plan Apochromatic 10x with a numerical aperture (NA) of 0.45 was used. A high-precision motorized stage was used to collect the large-scale 3D mosaics of each tissue section. The tissue sections were imaged on a high-resolution scientific complementary metal oxide semiconductor (sCMOS) camera (Zyla 4.2, 2.0 Andor, Oxford Instruments Company). The fusion (Andor, Oxford Instruments Company) and Image J/Fiji (v1.51s, Wayne Rasband, NIH, Bethesda, MD, USA) software were used for the acquisition and image processing.

### 4.7. Statistical Analysis

Data were expressed as the mean ± SEM. Statistical analysis was carried out using GraphPad Prism 6. The chi-square test, Student’s *t*-tests for dependent or independent samples and 2-way ANOVAs (area and stimulation as factors) for repeated measurements followed by Bonferroni’s post hoc test were used, as appropriate. Statistical significance has been set at the 95% confidence level (2 tailed).

## Data Availability

The data presented in this study are available on request from the corresponding author.

## Supplementary Materials

The following supporting information can be downloaded at: https://www.mdpi.com/article/10.3390/ijms24054891/s1.
