# Supplementary material for: Differential Modulation of Dorsal Raphe Serotonergic Activity in Rat Brain by the Infralimbic and Prelimbic Cortices
Source: Int J Mol Sci. 2023 Mar 3;24(5):4891. doi: 10.3390/ijms24054891 (PMC10003771; doi:10.3390/ijms24054891)
Supplement: Supplementary file 1 [file ijms-24-04891-s001.zip › ijms-2213620-supplementary.pdf]

Supplemental Informations

Differential modulation of dorsal raphe serotonergic activity in rat brain by the infralimbic and prelimbic cortices

Elena López-Terrones[1,2], Verónica Paz[1,2], Leticia Campa[1,2], Sara Conde-Berriozabal[4,3,5], Mercè Masana[4,3,5], Francesc Artigas[1,2,3], Maurizio S. Riga[6*]

[1]Depart. de Neurociències i Terapèutica Experimental, Institutd'InvestigacionsBiomèdiques de Barcelona (IIBB-CSIC) 08036 Barcelona, Spain.

[2]Centro de Investigación Biomédica en Red de Salud Mental (CIBERSAM), Instituto de Salud Carlos III, 28029 Madrid, Spain.

[3]Institut d'Investigacions Biomèdiques August Pi i Sunyer (IDIBAPS), 08036 Barcelona, Spain.

[4]Dept. de Biomedicina, Institut de Neurociències,Facultat de Medicina i Ciències de la Salut, Universitat de Barcelona, 08036 Barcelona, Spain.

[5]Centro de Investigación Biomédica en Red sobre Enfermedades Neurodegenerativas (CIBERNED), E-08036 Barcelona, Spain.

[6]Centro Andaluz de Biología Molecular y Medicina Regenerativa (CABIMER-CSIC), 41092 Sevilla, Spain.

*Correspondence: Maurizio S. Riga, Centro Andaluz de Biología Molecular y Medicina Regenerativa (CABIMER-CSIC), Av. Americo Vespucio 24, 41092 Sevilla, Spain. Email: mauririga@gmail.com.

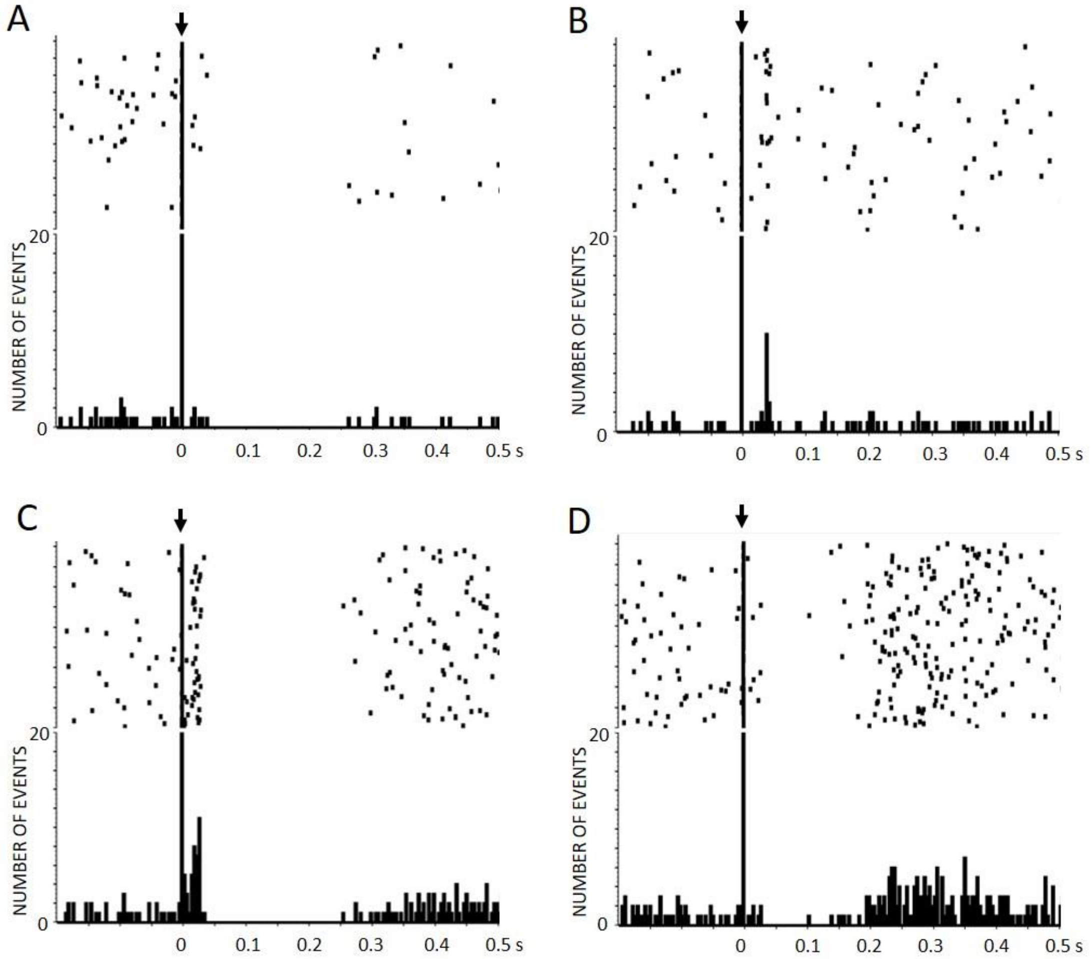

Supplemental Figure S1. Peristimulus-time histograms (PSTH) and raster plots showing representative examples of the different responses induced in DR serotonin neurons by IL and PrL stimulation at S1 (0.9 Hz, 0.2 ms, 1.7 mA). **(A)** Pure inhibitory response; **(B)** Pure ortodromic activation; **(C)** Ortodromic activations following by inhibitions (OA+I); **(D)** Inhibitions following by ortodromic activations (I+OA). Arrow mark stimulations.

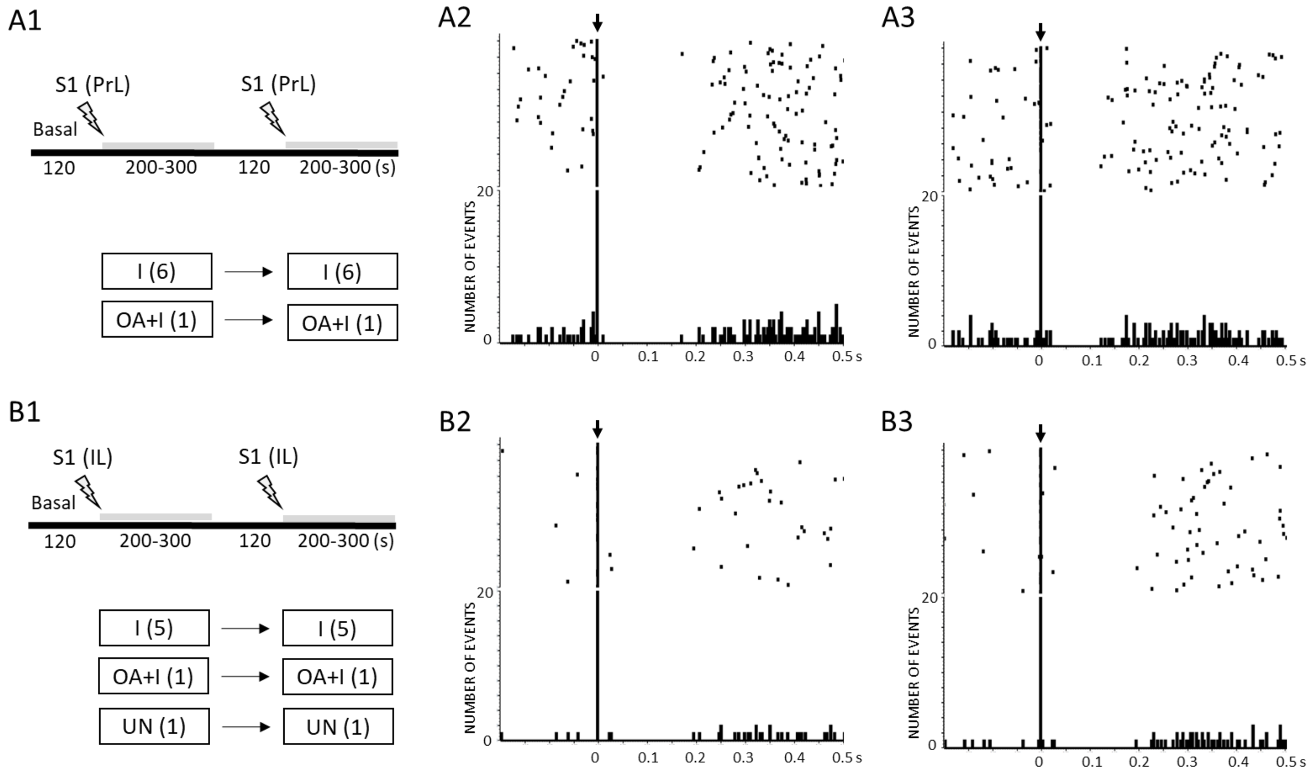

Supplemental Figure S2. A1 and **B1:** Schematic representation of the protocol used for studying the response of the same dorsal raphe (DR) 5-HT neurons to a second stimulation from the same site (prelimbic, PrL; infralimbic IL) using S1 stimulation settings (0.9 Hz, 0.2 ms, 1.7 mA). Note that all neurons maintained the response type during the 2nd stimulation from the same site. Abbreviations: I (inhibitions), OA+I (orthodromic activation followed by inhibitions), UN (unaffected). **A2** and **A3** show the response of a 5-HT neuron to PrL (1st) and PrL (2nd) stimulation, respectively. **B2** and **B3** show the response of a 5-HT neuron to IL (1st) and IL (2nd) stimulation, respectively.

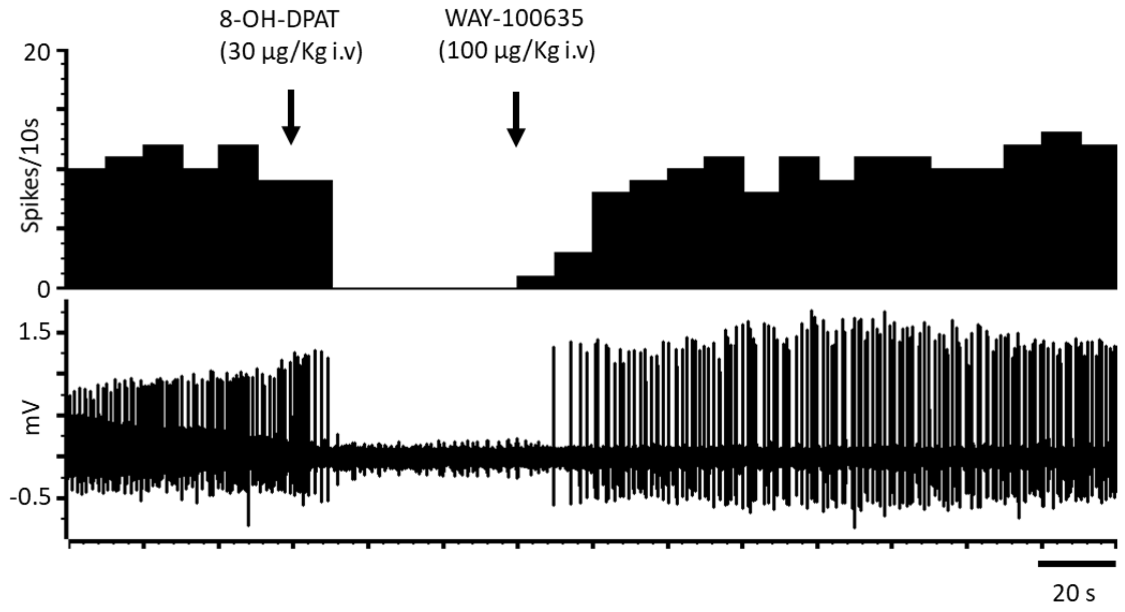

Supplemental Figure S3. Representative example of a serotoninergic neuron recorded from dorsal raphe (DR) nucleus. Note its constant firing properties(around 1 Hz) in basal conditions. Pharmacological identification was also performed by administrating the 5-HT_{1A}-R agonist 8-OH-DPAT and, subsequently the 5-HT_{1A}-R antagonist WAY-100635. As expected, 8-OH-DAP administration inhibited the firing properties of the recorded neuron and the blockade of 5-HT_{1A}-R induced by WAY-100635 reversed the effect.
